# Modeling Growth Kinetics, Interspecies Cell Fusion, and Metabolism of a Clostridium acetobutylicum/Clostridium ljungdahlii Syntrophic Coculture

**DOI:** 10.1128/mSystems.01325-20

**Published:** 2021-02-23

**Authors:** Charles Foster, Kamil Charubin, Eleftherios T. Papoutsakis, Costas D. Maranas

**Affiliations:** a Department of Chemical Engineering, The Pennsylvania State University, University Park, Pennsylvania, USA; b Department of Chemical and Biomolecular Engineering, The University of Delaware, Newark, Delaware, USA; c The Delaware Biotechnology Institute, Molecular Biotechnology Laboratory, University of Delaware, Newark, Delaware, USA; University of California, Berkeley

**Keywords:** clostridia, *Clostridium acetobutylicum*, *Clostridium ljungdahlii*, cell fusion, cell growth, community modeling, dynamic flux balance analysis, genome-scale metabolic modeling, metabolism, microbial consortia

## Abstract

Clostridium acetobutylicum and Clostridium ljungdahlii grown in a syntrophic culture were recently shown to fuse membranes and exchange cytosolic contents, yielding hybrid cells with significant shifts in gene expression and growth phenotypes. Here, we introduce a dynamic genome-scale metabolic modeling framework to explore how cell fusion alters the growth phenotype and panel of metabolites produced by this binary community. Computational results indicate *C. ljungdahlii* persists in the coculture through proteome exchange during fusing events, which endow *C. ljungdahlii* cells with expanded substrate utilization, and access to additional reducing equivalents from C. acetobutylicum-evolved H_2_ and through acquisition of C. acetobutylicum-native cofactor-reducing enzymes. Simulations predict maximum theoretical ethanol and isopropanol yields that are increased by 0.64 mmol and 0.39 mmol per mmol hexose sugar consumed, respectively, during exponential growth when cell fusion is active. This modeling effort provides a mechanistic explanation for the metabolic outcome of cellular fusion and altered homeostasis achieved in this syntrophic clostridial community.

**IMPORTANCE** Widespread cell fusion and protein exchange between microbial organisms as observed in synthetic C. acetobutylicum/*C. ljungdahlii* culture is a novel observation that has not been explored *in silico*. The mechanisms responsible for the observed cell fusion events in this culture are still unknown. In this work, we develop a modeling framework that captures the observed culture composition and metabolic phenotype, use it to offer a mechanistic explanation for how the culture achieves homeostasis, and identify *C. ljungdahlii* as primary beneficiary of fusion events. The implications for the events described in this study are far reaching, with potential to reshape our understanding of microbial community behavior synthetically and in nature.

## INTRODUCTION

The genus *Clostridium* is comprised of Gram-positive, rod-shaped, anaerobic bacteria with a unique diversity in metabolic functionality (1–3). *Clostridium* organisms are capable of consuming the vast majority of biomass-derived carbohydrates, collectively producing a broad range of fermentation products. Organisms within the genus identified as promising for chemical and biofuel synthesis have been categorized based on their fermentation product profiles and substrate utilization, and they generally fall into four broad and overlapping categories: solventogens, acetogens, cellulolytic organisms, and chain elongators (2, 4). This natural metabolic versatility has motivated the development of several *Clostridium* organisms as production platforms in both monocultures and cocultures. Solventogenic Clostridium acetobutylicum (*Cac*) has been a prominent industrial production platform for ABE (acetone-butanol-ethanol) fermentation for more than a century (5) and has been elevated into the exemplar butanol-producing organism (3, 4, 6–8). Many efforts have focused on improving productivity (9–21) or shifting fermentation product profile toward the IBE (isopropanol-butanol-ethanol) fermentation (22–24). Acetogens Clostridium ljungdahlii (*Clj*) and Clostridium autoethanogenum have both been explored for bioprocessing by exploiting their native ability to directly convert CO_2_ and CO to ethanol and acetate using the Wood-Ljungdahl pathway (WLP) (25–29). In addition, different autotrophic clostridia have been coupled with Clostridium kluyveri in synthetic cocultures to produce valuable medium-chain alcohols and fatty acids from syngas (30–32).

Complementary to experimental efforts aimed at developing these organisms into viable production platforms, computational genome-scale reconstruction and analysis efforts have been directed toward both understanding the metabolism of *Clostridium* organisms and redirecting it to achieve specific metabolic outcomes. Due to its long-standing industrial usage in the Weismann process, *Cac* is the most prominently modeled *Clostridium*. Several genome-scale reconstruction efforts have been undertaken to both understand metabolic phenotype (33–38) and engineer the overproduction of butanol (39). Genome-scale metabolic (GSM) model reconstruction and engineering of autotrophic *C. autoethanogenum* and *Clj* have been carried out to understand energy metabolism (40, 41), maximize autotrophic growth (42), maximize ethanol and 2,3-butanediol production from syngas (41, 43, 44), and understand proteome allocation (45). A community GSM model of a synthetic *C. autoethanogenum*/Eubacterium rectale coculture was also used to design a biocatalyst system with enhanced butyrate production (46).

Bolstering their potential as viable coculture fermentation platforms is the surprising observation that, in a glucose-fed—supplemented with small amounts of fructose—syntrophic coculture, the genetically distinct *Cac* and *Clj* fuse membranes and exchange their proteomes and RNA, resulting in a drastically different profile of fermentation products with enhanced substrate carbon recovery into products compared to physically separated cocultures (47, 48). *Cac* uses glucose, fructose, and other sugars to produce acetate, butyrate, butanol, acetone, ethanol, acetoin, CO_2_, and H_2_. *Clj* cannot use glucose (using only fructose among the common sugars) and cannot survive in a glucose-based culture medium in monoculture. However, in syntrophic coculture, it grows using CO_2_ and H_2_ released by *Cac*. When grown in monoculture in the basal medium of the coculture but supplemented with fructose and/or CO_2_/CO/H_2_, *Clj* produces only acetate and ethanol (48). In coculture, acetone was converted to isopropanol (2-propanol, which neither organism produces in monoculture), and acetoin to 2,3-butanediol (which *Clj* can in principle produce alone but did not in control cultures) (48). In coculture, 2,3-butanediol was formed without any detectable acetoin in the medium, thus suggesting direct transfer of acetoin from *Cac* to *Clj* (48). Furthermore, the large concentrations of 2-propanol produced in coculture combined with electron balances for the individual-species metabolism suggested direct electron transfer (48). Significantly, there was a larger fraction of substrate carbon converted into products with much lower levels of CO_2_ and H_2_ released. These phenotypes were abolished when the two organisms were separated by a permeable membrane (48).

In this study, we aim to gain insight into how the observed cell fusion/protein exchange events shape metabolism to enable the enhanced fermentation product yields. To this end, we first developed a growth kinetic model that accounts for the cell fusion events and the growth of both nonhybrid *Cac* and *Clj* as well as *Cac* and *Clj* cells in a hybrid state, that is, cells that contain proteins from both organisms. By introducing a single fusion parameter (characterizing the rate of cell fusion/proteome exchange in the coculture), we are able to recapitulate the growth kinetics observed experimentally and accurately predict the relative abundance of each organism through the stationary phase despite significant differences between the specific growth rates of each organism. We then couple the growth model with a dynamic multispecies metabolic modeling framework (DMMM) (49) to quantify how community metabolism changes as a result of the observed cell fusion events. When we considered our coculture growth model with both nonhybrid and hybrid GSM models for *Cac* and *Clj* in DMMM, approximately 10-fold increases in ethanol and 2,3-butanediol maximum theoretical titer and a 5-fold increase in isopropanol maximum theoretical yields (per unit of hexose sugar consumed) were observed after 33 h. Results agree qualitatively with experimental observations and point toward the expanded substrate utilization and cofactor regeneration capacity in hybrid *Clj* (compared to nonhybrid *Clj*) as a primary reason for both the persistence of *Clj* in the coculture at high abundance and the observed shift in fermentation product profiles and yields. Maximum *Cac* H_2_ evolution (and thus *Clj* H_2_ uptake) and unconstrained soluble carbon cross-feeding were required to achieve the observed maximum theoretical titers, indicating that the transfer of metabolic activity during cell fusion events and the cross-feeding of soluble carbon and reducing equivalents both play a role in shaping the observed coculture phenotype.

## RESULTS

### Growth model with cell fusion recapitulates hybrid cell abundance and predicts species relative abundance.

**FIG 1:**
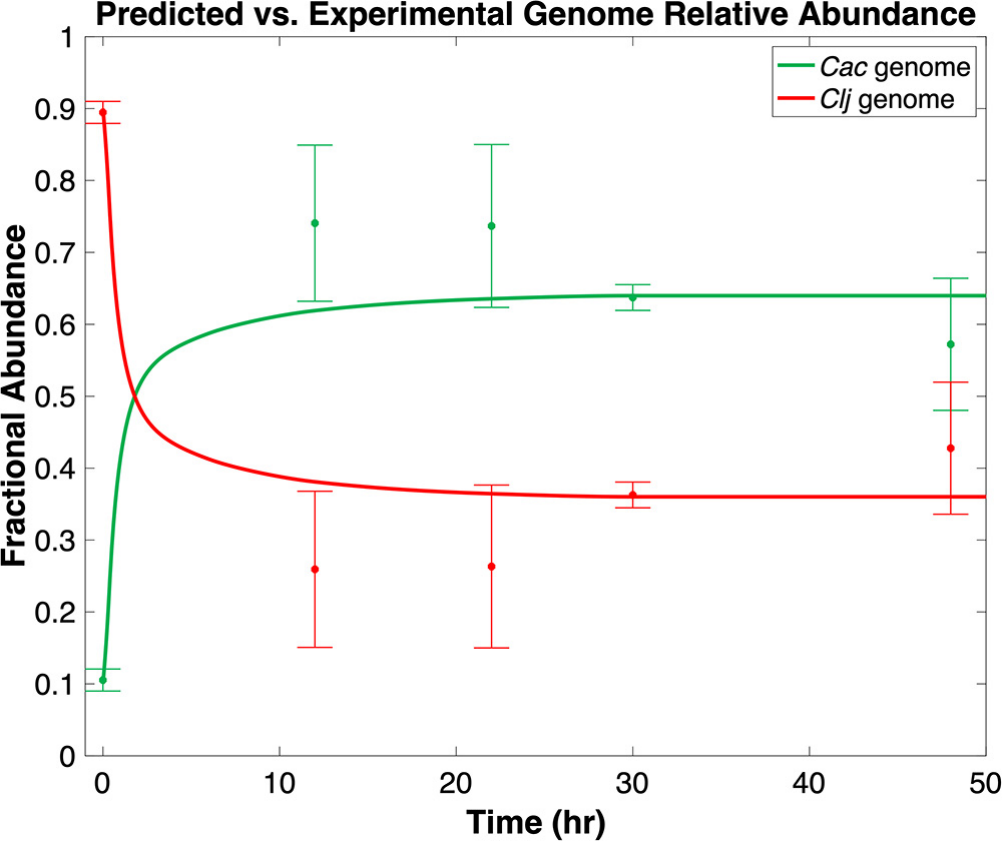
A comparison of the predicted time-dependent fractional abundance of *Cac* and *Clj* genome in coculture with experimental ranges. Error bars correspond to ±1 standard deviation from mean gene copy number measurements.

We fitted the growth model to the experimentally measured relative abundances of *Cac* and *Clj* cells over a 48-h culture. Figure 1 compares model predictions to experimental data. Relative abundances were calculated from genome copy number data (48). Consistent with experiments, initial conditions for the growth model ordinary differential equation (ODE) integration were defined as 10% nonhybrid *Cac* and 90% nonhybrid *Clj* (48). The growth model fusion parameter (*f*) which minimized the weighted squared Euclidian distance between model prediction and experimental measurements upon integration was selected for use. Standard deviations calculated from three replicate experimental measurements per data point were used to weight the objective function. The obtained value for *f* was 1.09 × 10^−12^ liter cells^−1^ h^−1^. The predicted relative abundance of each organism agreed with experimental relative abundances across the stationary phase and fell within a single standard deviation of experimental values for all data points except at the 12-h time point. In addition to predicting the organism abundances well, the growth model predicted both hybrid and nonhybrid *Cac* cells to grow similarly to nonhybrid *Cac* cells in monoculture despite continuously occurring fusion events. This suggests that despite proteome exchange, *Cac* growth phenotype is unchanged by fusion with *Clj*. This also suggests that fusion events prevent slower-growing *Clj* from being outcompeted by the faster-growing *Cac* in the culture (as happens when growth is simulated in the absence of fusion events). Thus, the primary metabolic shift caused by cell fusion/proteome exchange occurs in *Clj* cells. The trend of rapid increase in *Cac* abundance followed by more or less constant abundance of *Cac* and *Clj* across the remainder of the simulation is in agreement with experimental observations.

**FIG 2:**
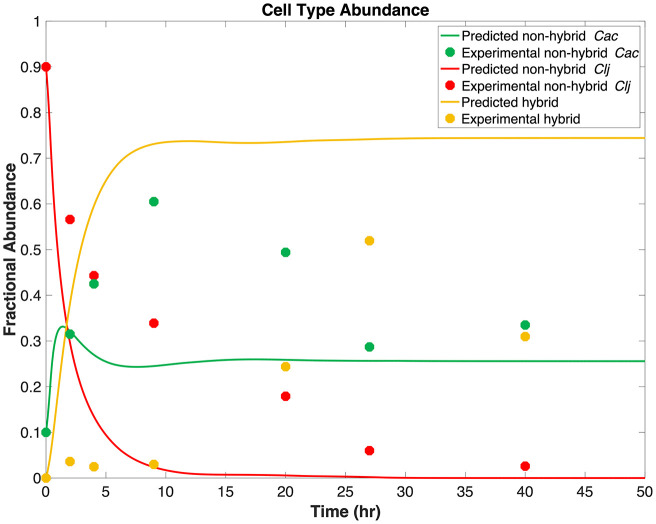
Comparison of the relative abundance of nonhybrid and hybrid cells in the coculture estimated by the growth model with experimentally observed abundances. The time interval in the figure (and used for the simulation) represents the time from inoculation (time = 0 h) through the end of the stationary phase (time = 48 h).

Figure 2 shows the predicted change in relative abundances of nonhybrid *Cac*, nonhybrid *Clj*, and total hybrid cells over time and compares those values to experimentally resolved (47) relative abundances. An initial lag in cell fusion was observed experimentally but fell outside the predictive capability of our model, as we chose to describe the system using a single fusion parameter value with a clear mechanistic meaning, rather than attempting to identify empirical correlations which could better match model predictions to experimental measurements. As *Clj* was transformed from nonhybrid to hybrid cells, the abundance of nonhybrid *Clj* rapidly declined while the abundance of hybrid cells rapidly increased. Nonhybrid *Cac* was predicted to grow rapidly upon inoculation, but after 1.4 h, predicted nonhybrid *Cac* cell relative abundance reached a maximum and decreased when hybrid surpassed nonhybrid *Cac* abundance. The rapid growth of nonhybrid *Cac* also ensured nonzero relative abundance during the stationary phase. The rapid increase in nonhybrid *Cac* relative abundance followed by a decrease in relative abundance agrees qualitatively with experimental measurements, as does the rapid decrease in relative abundance of nonhybrid *Clj* cells. However, the maximum nonhybrid *Cac* relative abundance is 27% less than the experimental maximum nonhybrid *Cac* relative abundance, and the maximum predicted hybrid cell relative abundance is 22% greater than the experimental maximum hybrid cell relative abundance. Because a single fusion parameter was used, the growth model was also unable to capture the initial lag in hybrid cell formation observed in experiments, and the maximum experimental nonhybrid *Cac* relative abundance was measured 7.6 h after the model predicted nonhybrid *Cac* relative abundance reached its maximum value. While Fig. 2 indicates that agreement with experiments is qualitative at best, a significant number of unlabeled cells across all measured time points were reported (more than 10% of cells were unlabeled across four of six time points) with the experimental flow cytometry results. In fact, a maximum of 48% of cells were unlabeled (did not fluoresce) at the 40-h time point. Therefore, these data were not considered for model training and were used only for comparative purposes to highlight where attention is needed from both experimentalists and modelers to better understand the mechanisms at play in this system. As previously noted (47), it is likely that cell fusion events and hybrid cells are even more abundant than what is captured through flow cytometry, which is in line with the higher abundance of hybrid cells predicted by the fusion/growth model.

**FIG 3:**
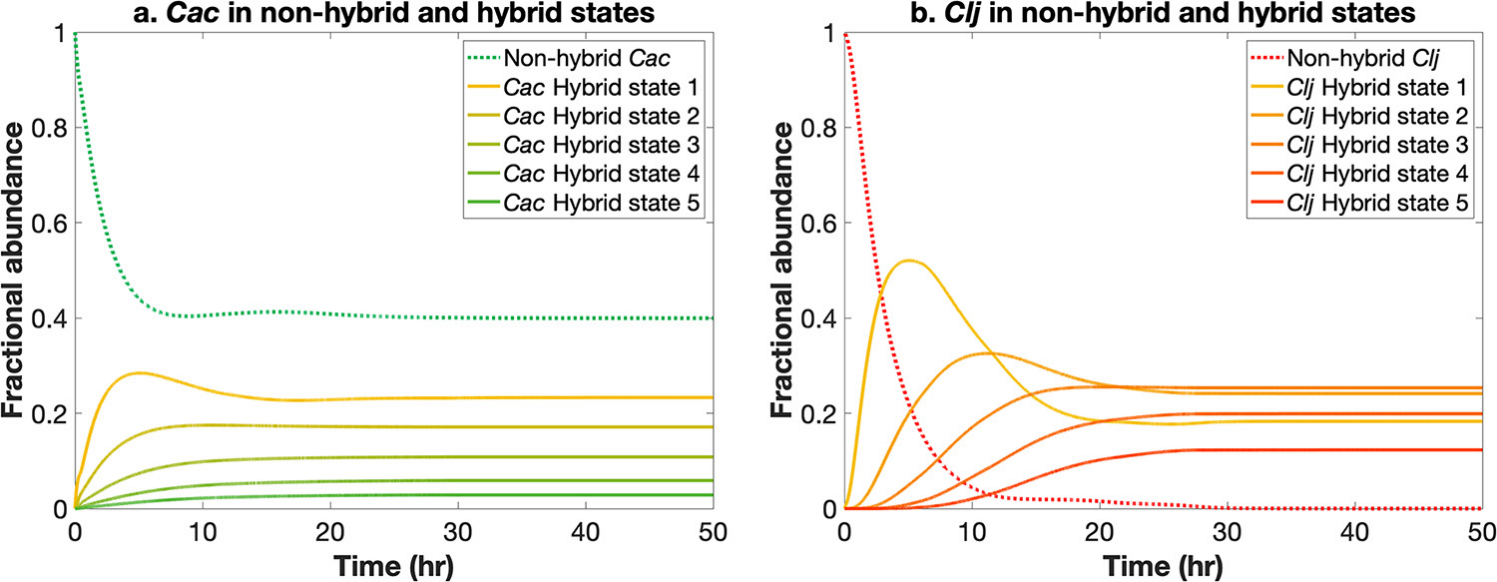
(a) Growth model-predicted *Cac* fractional abundance in nonhybrid and hybrid states. (b) Growth model-predicted *Clj* fractional abundance in nonhybrid and hybrid states.

Using the growth model defined for nonhybrid and hybrid state cells in equations 2 to 4, the relative abundances of nonhybrid and hybrid-state *Cac* and *Clj* cells in the coculture were predicted. Figure 3 shows the change in the relative abundances of nonhybrid and hybrid *Cac* and *Clj* cells over time. The faster-growing *Cac* cells reached steady state after approximately 30 h and were primarily in nonhybrid form (42% nonhybrid *Cac* at time = 30 h). Slower-growing *Clj* cells reached a steady state in relative abundance after approximately 35 h and were primarily in hybrid state (99% hybrid *Clj* at time = 35 h). The predominance of *Cac* in its nonhybrid form was due to a higher mid-exponential growth rate which ensured that the rate of cell fusion/proteome exchange did not exceed the growth rate of nonhybrid *Cac*. The faster equilibration of the *Cac* abundance was the result of a rapid decrease in *Cac* growth rate after its exponential phase (consistent with the onset of the solventogenesis and sporulation program). Note that after 15 h the *Cac* growth rate was an order of magnitude lower than that of *Clj*. The predicted dominance of *Cac* in its nonhybrid form and the rapid depletion of nonhybrid *Clj* are both in qualitative agreement with experimental flow cytometry results (47). Absolute and relative abundances of hybrid and nonhybrid cells across the growth model simulation are reported in Data Set S3 in the supplemental material.

### Hybrid model flux variability analysis reveals nonnative reactions contributing to growth phenotype.

Table 1 describes the active network of hybrid and nonhybrid GSM models used to simulate coculture metabolism and their observed maximum growth rates upon flux balance analysis (FBA). Reaction contents from iCAC802 and iJL680 were merged to create hybrid models, but organism-specific biomass equations were retained because significant DNA exchange was not observed experimentally in a single 40-h coculture fermentation (47). In order to understand how hybrid cell metabolism was affected by cell fusion events, we performed flux variability analysis (FVA) with hybrid and nonhybrid models to identify the set of nonnative reactions whose bounds did not span or contained zero (thus influencing growth rate) in each hybrid model under maximum growth conditions.

**TABLE 1:** Active metabolic network and maximum growth rate using 5 mmol gDW^−1^ h^−1^ fructose as the sole carbon source under nutrient-rich conditions for nonhybrid and hybrid cell models

Model	Unblocked reaction	Total metabolites	Source of biomass equation	Max growth rate (h^−1^)
*Cac* nonhybrid (iCAC802)	527	438	iCAC802	0.79
*Clj* nonhybrid (iJL680)	539	453	iJL680	0.22
*Cac* hybrid	764	496	iCAC802	0.81
*Clj* hybrid	713	579	iJL680	0.24

After removing thermodynamic infeasible and energy-generating cycles, a total of 120 *Cac*-specific metabolic reactions from iCAC802 were capable of carrying flux in the *Clj* hybrid cell model, and 55 native *Clj* reactions which were blocked in iJL680 became unblocked. The expanded activity resulted in a 4.5% increase in maximum growth rate compared to iJL680 when 5 mmol gram dry weight (gDW)^−1^ h^−1^ fructose was used as the sole carbon source. This increased growth rate was the result of only two necessarily active *Cac*-specific reactions: NADPH:NAD^+^ oxidoreductase (β-specific) and (*S*)-dihydroorotate:(acceptor) oxidoreductase. The expanded cofactor regeneration capacity afforded by NADPH:NAD^+^ oxidoreductase (β-specific) enabled hybrid *Clj* to supply additional reducing equivalents in the form of NADPH to fuel biomass precursor (fatty acid, amino acid, pantothenate, and coenzyme A) synthesis pathways and increase growth. In the absence of an external H_2_ supply, the NADP^+^ reduction critical for supplying electrons to biomass precursor synthesis in nonhybrid *Clj* is dependent upon the availability of an equal ratio of NADH and reduced ferredoxin and is catalyzed by ferredoxin:NADP reductase (45). Because NADPH:NAD^+^ oxidoreductase (β-specific) is dependent upon a single NADH, to reduce a single NADP^+^ molecule to NADPH, its presence increased net NADPH availability from 6.9 mmol gDW^−1^ h^−1^ in iJL680 to 7.0 mmol gDW^−1^ h^−1^ in hybrid *Clj* under maximum growth conditions. (*S*)-Dihydroorotate:(acceptor) oxidoreductase is menaquinone dependent in *Clj* (45), but NAD^+^ dependent in *Cac* (35). Inclusion of *Cac* (*S*)-dihydroorotate:(acceptor) oxidoreductase replaced menaquinone dependence with a reaction which regenerates NADH in the pyrimidine synthesis pathway and decoupled the conversion of orotate to *S*-dihydroorotate from fumarate reductase activity, which is otherwise needed to oxidize menaquinone. This enabled an increase in UMP availability for nucleotide metabolism from 0.028 to 0.031 mmol gDW^−1^ h^−1^ to marginally improve growth rate. While iJL680 could not grow when glucose was substituted for fructose as the sole carbon source (as *Clj* does not natively produce glucose transporters), the hybrid *Clj* model predicted a growth rate identical to that observed with fructose as the sole carbon source and identical *Cac*-native reactions contributing to the growth phenotype. Reactions converting glucose to fructose 1,6-bisphosphate (fdp) were necessarily active upon glucose substitution. However, because the net cofactor demand for conversion of glucose to fdp is identical to the requirement for converting fructose to fdp, the predicted growth rates were identical.

Upon inclusion of iJL680 reactions in the *Cac* hybrid model, and after removing thermodynamic infeasible and energy-generating cycles, 159 previously blocked reactions in iCAC802 and 85 reactions in iJL680 became capable of carrying flux. The expanded active network of hybrid *Cac* yielded a 2.5% increase in maximum growth rate compared to iCAC802 with 5 mmol gDW^−1^ h^−1^ of either glucose or fructose as the sole carbon source. This was the result of *Clj*-specific phosphoserine transaminase activity. In *Cac*, l-serine is synthesized from glycine (generated primarily through threonine degradation), with a carbon donated from 5,10-methylenetetrahydrofolate. Inclusion of phosphoserine transaminase completes the pathway which synthesizes serine directly from 3-phosphoglycerate (3-pg). Direct l-serine synthesis from 3-pg rather than as a by-product of folate biosynthesis and threonine degradation increased l-serine production by 0.14 mmol gDW^−1^ h^−1^ in hybrid *Cac*, consistent with the observed improvement in growth efficiency. Thus, while the majority of nonnative metabolic reactions merged into hybrid models are either replicated or unnecessary to support extra growth, a small subset of reactions help to improve growth rate in each organism upon cell fusion/proteome exchange. In *Clj*, this is achieved primarily through more efficient electron transfer and cofactor regeneration, while in *Cac*, this is achieved through l-serine synthesis directly from glycolytic intermediate 3-pg. Flux variability analysis results for each hybrid and nonhybrid metabolic model are provided in Data Set S2.

### Hybrid model DMMM simulation recapitulates coculture phenotype and sheds light on cross-feeding.

Two scenarios were considered when simulating coculture metabolism with DMMM. In the first scenario (here referred to as the nonfusing case), only nonhybrid metabolic models and standard Monod growth kinetics were used to describe metabolism and growth. In the second scenario (here referred to as the cell fusing case), both hybrid and nonhybrid metabolic models were used with the growth model developed in this study to describe metabolism, cell fusion, and growth. In both scenarios, culture substrate consumption was constrained by experimental fermentation profiles as outlined in Materials and Methods. In the nonfusing case, CO_2_ and H_2_ cross-feeding were *Clj*’s primary source of carbon and external reducing equivalents needed for growth. Results indicate that these activities are insufficient to support the observed *Clj* growth. When CO_2_ and H_2_ cross-feeding was maximized, within 8 h *Clj* made up less than 1% of total biomass. Furthermore, except for acetone and l-lactate, maximum theoretical extracellular titers predicted by the simulation without fusing events were less than the experimentally reported titers, which implies that additional sources of carbon and reducing equivalents must be accessible to *Clj* in the coculture. In contrast, under the biomass maximization assumption the maximum predicted l-lactate titer was over 20-fold greater than the experimental titer of 0.03 g/liter (possibly because l-lactate dehydrogenase is activated by fructose 1,6-bisphosphate in *Cac*). Because acetone is a fermentation product of *Cac* exclusively, the increased *Cac* abundance predicted by the nonfusing case led to an increase in the acetone maximum theoretical titer, which was 1.1 g/liter greater than the experimental titer. However, the sum of the maximum theoretical yields for isopropanol and acetone was 50% less than the combined experimental acetone and isopropanol yields. This indicates that the nonfusing case is incapable of delivering enough flux toward two- or three-carbon fermentation products for even qualitative agreement with experiments.

**FIG 4:**
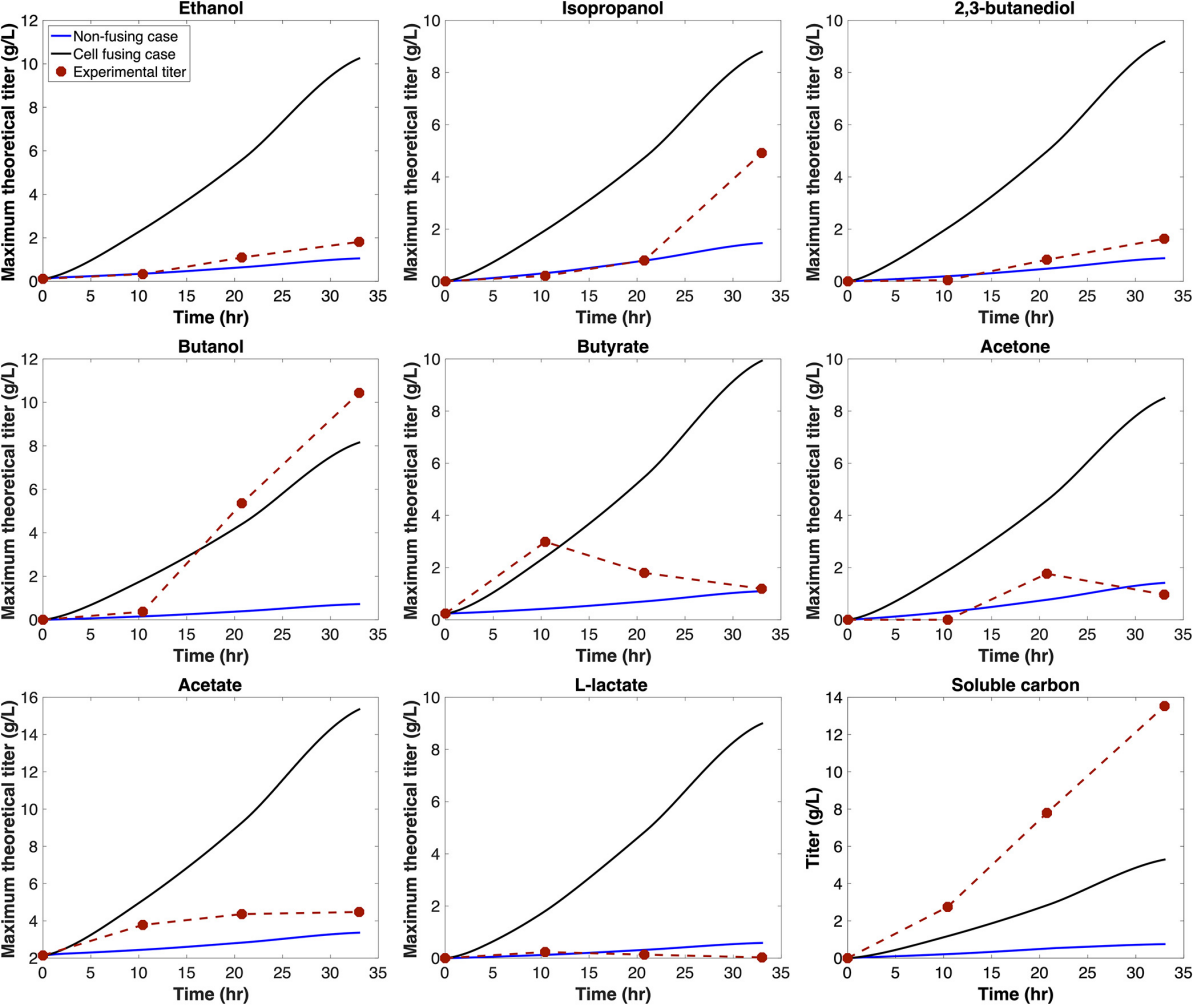
Comparison of maximum theoretical extracellular titers predicted by DMMM across 33-h time interval by the nonfusing case and cell fusing case alongside experimentally measured fermentation profiles for ethanol, isopropanol, 2,3-butanediol, butanol, butyrate, acetone, acetate, l-lactate, and total soluble carbon.

In contrast, *Clj* persisted in the cell fusing case, and maximum theoretical extracellular titers at the 33-h mark were increased by between 4.59- (acetate) and 15.40-fold (l-lactate) over the nonfusing case. *Clj* was able to persist because the hybrid *Clj* model contained glucose transporters, giving *Clj* access to the culture’s primary carbon source. Predicted maximum titers also exceeded experimental titers for all fermentation products except butanol. The cell fusing case delivered enough carbon toward isopropanol to exceed summed experimental acetone and isopropanol yields, and enough carbon was directed toward either acetate or ethanol to exceed summed experimental two-carbon fermentation product yields after 33 h of simulation time. Figure 4 compares the difference in time-varying maximum theoretical extracellular titers predicted by nonfusing and cell fusing cases alongside experimental measurements for eight fermentation products and total soluble carbon upon maximizing theoretical biomass yields across 33 h. We define soluble carbon as all atomic carbon contained in extracellular fermentation product pools. The results demonstrate increased titer/yield when fusion events are present in the simulation and better qualitative agreement with experimental observations. All yields are calculated for a basis of per millimole hexose sugar consumed.

While maximum theoretical titers predicted by the cell fusing case agree qualitatively with experimental results, demonstrating the need for hybrid metabolic models and inclusion of a kinetic description of cell fusion in the growth model to explain coculture phenotype, results fall short of quantitative agreement. Under the maximum growth assumption, soluble carbon titer falls below experimental values at every time point across the simulation, and at the 33-h time point the cell fusing case had produced 5.3 g/liter of soluble carbon, while the experimental soluble carbon titer was 13.5 g/liter. This likely results from events outside the DMMM predictive scope. For example, DMMM assigns maximum growth as an objective, but *Cac* cells do not maximize growth and grow slowly (if at all) during solventogenesis and sporulation (50, 51). Fermentation profiles indicate *Cac* solventogenesis begins at approximately the 11-h time point (marked by the onset of butyrate consumption and enhanced butanol production in the experimental fermentation product profiles).

**FIG 5:**
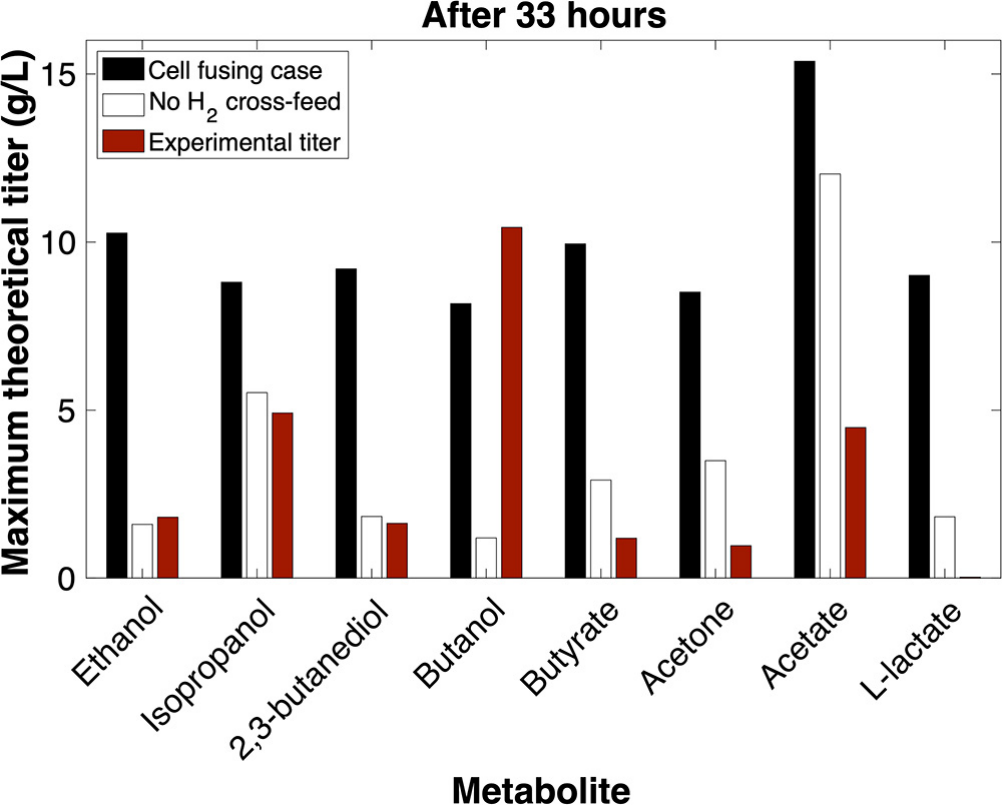
Maximum theoretical fermentation product extracellular titers predicted by the cell fusing case (with H_2_ cross-feeding) and without H_2_ cross-feeding at the 33-h time point in the DMMM simulation alongside experimentally measured titers.

The ability of the cell fusing case to better recapitulate experimental fermentation titers is partially caused by the inclusion of hybrid models. However, H_2_ cross-feeding also plays a role in increasing maximum theoretical fermentation product titers in the simulation. To better understand how H_2_ cross-feeding impacts simulation results, we performed an additional simulation (here referred to as no H_2_ cross-feed) in which we estimated maximum theoretical fermentation product titer when H_2_ cross-feeding from *Cac* to *Clj* was blocked. Figure 5 compares maximum theoretical titers predicted by the cell fusing case and no H_2_ cross-feed after 33 h. Acetate was the fermentation product with the least variability between the two scenarios. When interspecies H_2_ exchange was blocked, the maximum theoretical acetate titer decreased by 21%. Acetate synthesis is not dependent upon reduced cofactors. Therefore, decreased *Clj* abundance in the absence of cross-feeding was the only limitation to acetate formation, and not the availability of reducing equivalents, as was the case with other fermentation products. Isopropanol was the only other fermentation product for which, under the no H_2_ cross-feed scenario, maximum theoretical titer did not decrease by more than 58%. Isopropanol was exported by both hybrid *Cac* and hybrid *Clj*. Hybrid *Clj* converted acetone cross-fed from nonhybrid *Cac* to isopropanol whereas hybrid *Cac* converted cross-fed acetate from hybrid *Clj* to isopropanol, enabling a combined maximum titer within 37% of the cell fusing case. However, under no H_2_ cross-feed, the maximum theoretical titer exceeded experimental titer by only 0.6 g/liter. For ethanol, H_2_ cross-feeding was required to ensure that maximum theoretical titer after 33 h exceeded experimental titer. Even though the maximum theoretical butanol titer for the cell fusing scenario fell below the experimental value, upon H_2_ cross-feeding the maximum theoretical titer rose from 9.2 g/liter below the experimental titer to within 2.3 g/liter. The energy and reducing equivalent cost of acetate consumption was considerable, thus limiting acetate consumption and conversion to alcohol fermentation products.

**FIG 6:**
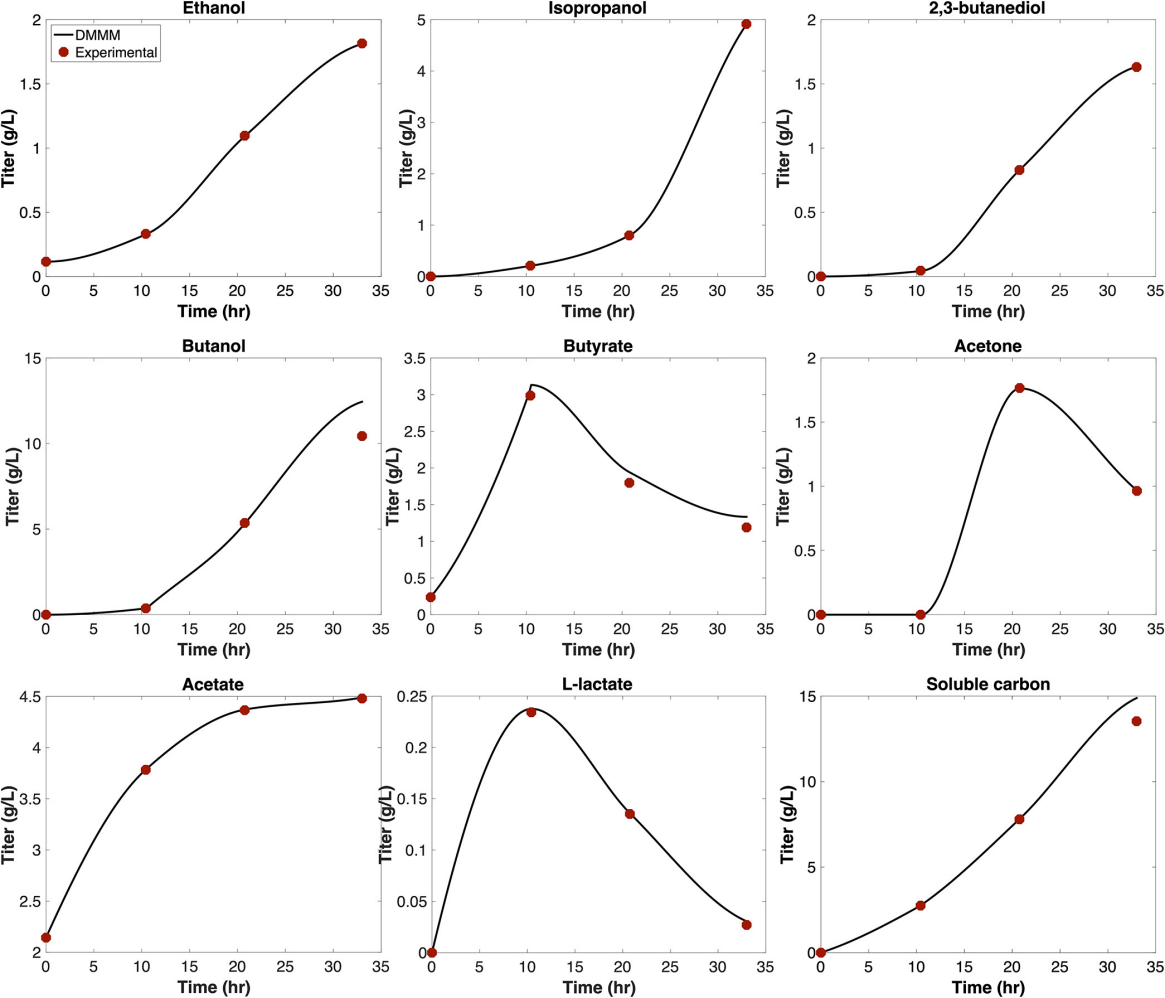
Comparison of predicted and experimental extracellular fermentation product titers in DMMM simulation with lower bounds on *Cac* and *Clj* growth rate relaxed to 56% and 5% of the theoretical maximum, respectively, during the first 10.4 h of the simulation and lower bounds on *Cac* relaxed to 35% of the theoretical maximum over the 10.4- to 33-h time interval. Substrate utilization was constrained according to species/cell-type relative abundances predicted by the growth model. Upper and lower bounds on fermentation product exchange fluxes were placed according to experimental production rates when fermentation products were produced during the culture. Lower bounds were placed on fermentation product uptake according to experimental consumption rates when fermentation products were observed to be consumed in experiments.

With the mechanistic details of how cell fusion enables metabolic shifts in coculture phenotype, we next sought to identify how we must constrain our model to enable quantitative agreement with the experimental fermentation product profiles, since multiple growth phases for both organisms in this system are known to deviate from maximum growth (50–52). Towards this end, we first predicted species/hybrid state cell abundances using our growth model in the absence of metabolic models. We then constrained substrate utilization by each organism/cell type in the metabolic simulation using the relative abundances of each organism/cell type predicted by our growth model. To achieve consistency with experimental soluble carbon yield during the first 10.4 h of the simulation, we set *Clj* growth rate to 5% of the theoretical maximum value. This relaxation is consistent with a *Clj* lag phase. In addition, the *Cac* growth rate was set to 56% of the theoretical maximum value during the first 10.4 h of the simulation. In the absence of this stipulation, the soluble carbon titer was 69% less than the experimentally reported titer after 10.4 h. Consistency with *Cac* solventogenesis and sporulation program was achieved by further reducing *Cac* growth rate to 35% of the theoretical maximum over the 10.4- to 33-h time interval. During the same time span, *Clj* was assumed to maximize growth, and growth was constrained to be within 1% of its theoretical maximum value. Across the 10.4- to 33-h time interval, we also allowed *Cac* and *Clj* to consume fermentation products in accordance with experimental observations. Table 2 shows the percent optimality choices, corresponding growth phases, and fermentation products consumed for each organism during three different time spans. Hybrid and nonhybrid *Cac* consumed butyrate and lactate during solventogenesis/sporulation programming, with the lower bound on rate of consumption of either compound defined according to rate of consumption calculated from interpolated fermentation profiles. Hybrid and nonhybrid *Clj* were allowed to consume acetone during the 21- to 33-h time interval to maintain consistency with experimental observations, with lower bounds on the rate of consumption placed according to the rate of consumption calculated from interpolated fermentation profiles. Figure 6 depicts a comparison of predicted fermentation profiles when a single FBA calculation was performed at each time step across a 33-h DMMM simulation with the abovementioned growth rate relaxations and added fermentation product uptake constraints. Except for butanol and butyrate, predicted fermentation profiles precisely match experimental measurements. Predicted extracellular butanol and butyrate titer deviated by at most 2.0 and 0.81 g/liter, respectively, from experimental titers between the 10.4- and 33-h time points. This result suggests that upon updating the optimality criteria of both *Cac* and *Clj* according to the growth stage, the modeling framework developed in this study accurately captures the experimentally observed fermentation profiles. Temporal maximum theoretical titers for the cell fusion, no cell fusion, and no H_2_ cross-feeding cases and the fermentation profiles estimated with added constraints to match experimental profiles are provided in Data Set S4.

**TABLE 2:** Growth efficiency and substrate utilization assumptions for *Cac* and *Clj* made to achieve consistency with experimental fermentation product profiles

Time interval (h)	Organism	Corresponding growth stage	Growth efficiency	Fermentation product(s) consumed
0–10.4	*Cac*	Exponential	0.56	
	*Clj*	Lag	0.05	
10.4–21	*Cac*	Solventogenesis/sporulation	0.35	Butyrate, lactate
	*Clj*	Exponential	1	
21–33	*Cac*	Solventogenesis/sporulation	0.35	Butyrate, lactate
	*Clj*	Exponential	1	Acetone

## DISCUSSION

In this study, we deployed a novel kinetic growth model and unique hybrid cell metabolic models within the DMMM framework to explore causes of the unexpectedly high fermentation product yields and persistence of *Clj* at high abundance in the *Cac*/*Clj* coculture. In the absence of a rate expression describing cell fusion and growth of hybrid state cells, a standard Monod growth kinetic model was insufficient to explain culture behavior. By adding rate expressions for cell fusion and hybrid cell population growth to our growth model, we were able to accurately predict species relative abundance through the stationary phase. By deploying our growth model within DMMM with novel hybrid metabolism genome-scale models, we have demonstrated the need for both to explain the coculture phenotype, as *Clj* is needed in high abundance to maximize fermentation yields but is outcompeted by *Cac* when it lacks glucose transporters acquired through cell fusion/protein exchange.

Although results largely agree with experiments, our model’s underprediction of soluble carbon titer indicates that there is room to constrain the model further to more accurately predict the coculture phenotype. The maximum growth assumption limits FBA scope in systems which do not tend to evolve toward biomass maximization (53). Other objectives, such as maximum ATP production (54, 55), minimum nutrient uptake (56), or maximization of growth and metabolite production (57), have been used within FBA to improve predictions in systems that do not tend to maximize biomass formation. In the *Cac*/*Clj* syntrophy, cells grow below the theoretical maximum yields during *Clj* lag (52), *Cac* solventogenic (50), and *Cac* sporulation (51) phases. Updating the optimality criteria allowed the model to allocate more carbon toward fermentation products to reach quantitative agreement with experimental extracellular titer measurements. Manually adjusting constraints to accommodate metabolic shifts in which the culture consumes fermentation products or recasting the problem in the form of a least-squares optimization which minimizes the difference between predicted and experimental extracellular titers also helps to improve prediction accuracy. Experimental fermentation profiles indicate two such events are present in this system. The first occurs at approximately the 11-h mark and corresponds to the solventogenic shift and onset of sporulation program in *Cac* (characterized by butyrate consumption and increased butanol excretion, and identical to the shift observed in *Cac* monoculture). The second metabolic shift occurs at approximately the 21-h mark. At this point, acetone accumulated in the medium is consumed, and isopropanol production increases dramatically. This shift was not observed in *Clj* monoculture because *Clj* does not produce acetone. Therefore, in addition to relaxing the lower bound of growth in each organism independently according to observed shifts in fermentation product profiles, better agreement with experimental soluble carbon titer was achieved by constraining fermentation product consumption and excretion in accordance with known metabolic shifts at time points inferred from fermentation product profiles.

In a broader context, the implications of this study in explaining how microbial communities achieve homeostasis are far reaching, with the potential to reshape our understanding of syntrophic, multimicroorganism systems. The striking difference between the behavior of simulations with and without hybrid cell models points to a need for reexamination of the potential for cell fusion and chemotaxis mechanisms in bacteria when modeling microbial consortia in order to reliably predict community behavior. The cell fusion events described in this study achieve large-scale protein exchange between bacteria, and allow for the persistence of *Clj* at a relative abundance that would be otherwise impossible. Because mechanistic interactions of microbial consortia are notoriously difficult to study (58), it is possible that this type of event is more widespread than we know, and a reason for the inexplicable persistence of slow-growing organisms at high relative abundances in other microbial communities. Persistence of slow-growing organisms in microbial communities is often attributed to extracellular polymeric substance excretion/biofilm formation (59), but this is not the case in the liquid culture examined in this study. The findings presented here point to a number of distinct metabolic advantages gained by *Clj* as the primary reason for cell fusion and pinpoint specific activities which enable the observed growth profiles and fermentation yields. However, the mechanistic details of how *Clj* is able to fuse membranes with *Cac* and extract the cytosolic material it needs to grow more efficiently remain unknown. A better understanding of this process could inform how synthetic cocultures should be designed for bioproduction in the future.

## MATERIALS AND METHODS

### Experimental batch fermentation data.

Batch fermentation data were obtained from the work of Charubin and Papoutsakis (48). Batch culture initial loading conditions were as follows: 5 g/liter fructose and 80 g/liter glucose supplemented with Turbo *Clostridium* growth medium (CGM) comprised of 1.0 g/liter KH_2_PO_4_, 1.25 g/liter K_2_HPO_4_, 1.0 g/liter NaCl, 0.01 g/liter MnSO_4_·H_2_O, 0.348 g/liter MgSO_4_, 0.01 g/liter FeSO_4_·7H_2_O, 2.0 g/liter asparagine, 5.0 g/liter yeast extract, 2.0 g/liter (NH_4_)_2_SO_4_, 2.46 g/liter sodium acetate, 0.20 mg/liter Na_2_WO_4_·2H_2_O, 0.02 g/liter CaCl_2_·2H_2_O, 4.0 mg/liter 4-aminobenzoic acid, 10 ml of 100× trace element solution, and 10 ml of 100× Wolfe’s vitamin solution. Coculture samples at approximate 12-h intervals through 48 h (corresponding to late stationary phase of the culture) were used for growth kinetic model training and validation purposes. Optical density measurements at a wavelength of 600 nm (OD_600_) were used to estimate specific growth rate of each organism required for the growth model. *Cac* and *Clj* genome copy number were used to estimate the relative abundance of each species in coculture and were determined using quantitative PCR (qPCR) (48). The relative abundance of hybrid cells, nonhybrid *Cac* cells, and nonhybrid *Clj* cells was determined using flow cytometry and florescence microscopy (47). Extracellular glucose, fructose, lactate, acetate, butanol, butyrate, acetone, acetoin, ethanol, isopropanol, and 2,3-butanediol titer measurements obtained using high-pressure liquid chromatography (HPLC) were used for metabolic model comparison and analysis (48). All experimental data used in this study are provided in Data Set S1 in the supplemental material.

10.1128/mSystems.01325-20.1 DATA SET S1 Experimental data used in this study, including genome copy number, coculture, *Cac* and *Clj* optical density measurements, flow cytometry data, and coculture fermentation product profiles. Download Data Set S1, XLSX file, 0.02 MB. Copyright © 2021 Foster et al. 2021 Foster et al. https://creativecommons.org/licenses/by/4.0/ This content is distributed under the terms of the Creative Commons Attribution 4.0 International license.

### Kinetic description of *Cac*/*Clj* coculture growth with cell fusion events.

Monod growth kinetics were adopted to describe the growth of both nonhybrid and hybrid *Cac* and *Clj* cells in the coculture. Specific growth rates (parameters in the growth model) for hybrid and nonhybrid cells were estimated from monoculture OD_600_ of each species that were inoculated under initial conditions similar to the coculture (48). To ensure linearity between adjacent points of the OD_600_ curve (and thus validity of Monod growth kinetics), p-chip interpolation (60) was performed before calculating time-dependent specific growth rate. Specific growth rate was calculated over 0.1-h intervals across 48 h (through stationary phase) according to equation 1 (61).

(1) μn=ln⁡(OD600nOD600n−1)tn−tn−1

where *n* are integer values on the interval [1, 481], *t_n_* is the time at *n* time point, μ*_n_* is the growth rate over the *t_n_*_−1_ to *t_n_* time interval, and ${\text{OD}}_{600}^{n-1}$ and ${\text{OD}}_{600}^{n}$ are the beginning and ending interpolated OD_600_ values over the *n*−1 to *n* time interval.

Four key assumptions were made in the kinetic description of coculture growth and cell fusion:

1 Fusion events occur only between nonhybrid cells.
2 Because growth rates for individual hybrid states are indistinguishable in the coculture, for each organism and hybrid state, the growth rate used corresponds to the time-dependent specific growth rate of the organism in monoculture with identical initial conditions calculated according to the interpolated monoculture OD_600_ curves.
3 No genomic DNA is exchanged during cell fusion events.
4 A total of five doubling times are required for cells that have exchanged proteins but retained their genetic identity to fully dilute all nonnative protein and revert back to nonhybrid cells. Each doubling time interval is characterized by a distinct cell population with a fractional abundance of nonnative proteins that decreases progressively as the number of doubling times since cell fusion increases. Five doubling times were chosen to ensure the model could capture the effect of hybrid cell recycling on nonhybrid cell abundance at later time points.

The system of ordinary differential equations (ODEs) describing the change in abundance with respect to time of all nonhybrid and hybrid state cells in the coculture system (and thus coculture growth) is represented by equations 2 to 4. The system accounts for two distinct cell types: (i) nonhybrid cells that have not exchanged proteins (nonhybrid *Cac* and nonhybrid *Clj*) and (ii) hybrid cells that have exchanged proteins but retained their genetic identities as either *Cac* or *Clj*. A single cell fusion parameter (*f*) was introduced to describe the rate of cell fusion/proteome exchange.

(2) $$\frac{dX_{0}^{k}}{\mathrm{dt}}=\mu_{0}^{k}X_{0}^{k}-\underset{k^{\text{′}}=\mathrm{Cac}\text{ or }\mathrm{Clj}}{\prod }fX_{0}^{k^{\text{′}}}+\mu_{5}^{k}X_{5}^{k},k=\mathrm{Cac}\text{ or }\mathrm{Clj}$$

(3) $$\frac{dX_{1}^{k}}{\mathrm{dt}}=\underset{k^{\text{′}}=\mathrm{Cac}\text{ or }\mathrm{Clj}}{\prod }fX_{0}^{k^{\text{′}}}-\mu_{1}^{k}X_{1}^{k},k=\mathrm{Cac}\text{ or }\mathrm{Clj}$$

(4) $$\frac{dX_{l}^{k}}{\mathrm{dt}}=\mu_{l-1}^{k}X_{l-1}^{k}-\mu_{l}^{k}X_{l}^{k},k=\mathrm{Cac}\text{ or }\mathrm{Clj},l=2,\ldots ,5$$

Equations 2, 3, and 4 model the growth of nonhybrid cells, hybrid cells in the first hybrid state, and hybrid cells at all other hybrid states, respectively. When integrated with respect to time, this system of ODEs predicts the dynamic coculture species/hybrid state composition. In these equations, superscripts *k* and *k*′ both correspond to the set of organisms in the coculture (i.e., *Cac* and *Clj*). Subscript *l* corresponds to the hybrid state, with a value of 0 representing nonhybrid cells, and values between 1 and 5 representing hybrid states after 1, 2, 3, 4, or 5 doubling times. Each of them contains decreasing amounts of the nonnative protein as, in the absence of replenishment through DNA transcription and translation, the abundance of nonnative protein in a hybrid cell is halved upon cell division. Although RNA is also exchanged between the two microbes, the short half-life of mRNA would not allow passing functional mRNA from the mother cell to daughter cells upon cell division. Parameters in the formulation include $\mu_{l}^{k}$, which is the specific growth rate of organism *k*; hybrid state *l*, which is time dependent and calculated using equation 1; and fusion parameter *f*. The fusion parameter is the rate constant relating the rate of fusion/proteome exchange to the abundance of nonhybrid *Cac* and nonhybrid *Clj* cells. We assume that fusing events are first order with respect to both nonhybrid *Cac* and nonhybrid *Clj* abundances. Dependent variables in the system of equations include $X_{l}^{k}$, which is the abundance of nonhybrid cells (if *l* = 0) or hybrid cells at different hybrid states (if *l* = 1, 2, 3, 4, or 5) of organism *k*.

**FIG 7:**
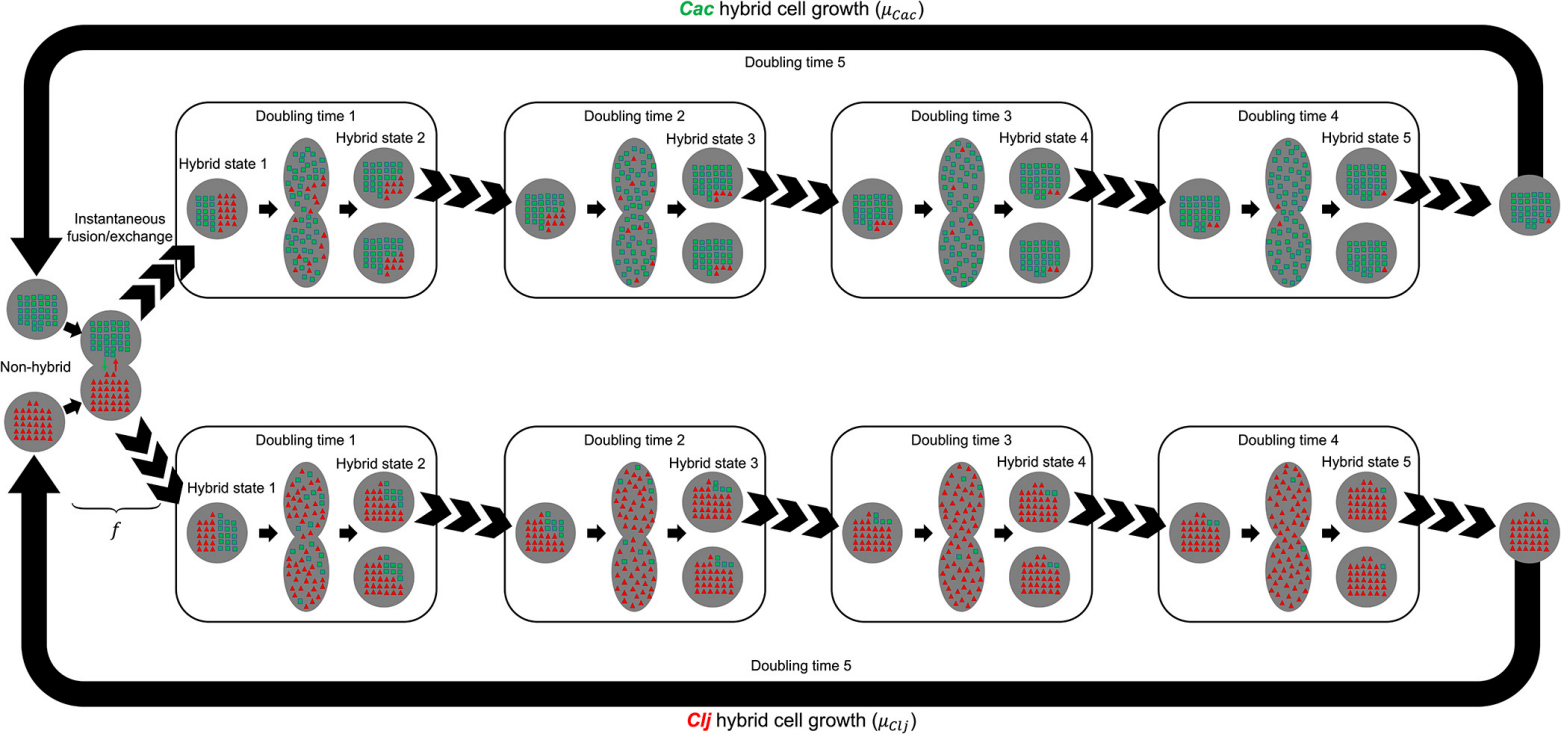
Nonnative protein dilution during cell growth. Modeling framework assumes six discrete cell populations of each organism corresponding to cells with various amounts of nonnative proteins. Each hybrid state corresponds to cells that have grown for 1, 2, 3, 4, or 5 doubling times since cell fusion/protein exchange. Nonnative proteins become more dilute as hybrid state increases, and cells at each hybrid state grow into cells at the next hybrid state. It is assumed that five doubling times are required to completely dilute all nonnative protein from cells. Thus, cells in hybrid state 5 grow into nonhybrid cells. Green squares represent *Cac*-native proteins, while red triangles represent *Clj*-native proteins.

Equation 2 contains three terms which contribute to the overall change in abundance of nonhybrid cells. The first term on the right-hand side of the expression corresponds to the Monod equation for growth of nonhybrid species. The second term quantifies the rate of fusion/exchange of protein events, with rate constant *f* multiplying nonhybrid *Cac* and nonhybrid *Clj* abundances. The third term in equation 2 is the Monod equation for growth of hybrid cells after the fifth doubling time, which we define as the point at which nonnative proteins in hybrid cells have fully diluted and the hybrid cell population has essentially reverted to the nonhybrid originating cell population. Equation 3 describes the change in the hybrid cell abundance at the first hybrid state (*l* = 1). This set of hybrid cells contain 50% of their own protein and 50% of proteins from the other organism. Similarly, equation 4 quantifies the change in hybrid cell abundances at *l* = 2, 3, 4, and 5 hybrid states. Figure 7 depicts the dilution of nonnative protein during cell culture growth across the five hybrid states.

### Genome-scale metabolic models.

GSM models of nonhybrid cells were built using the recent genome-scale reconstructions of *Cac* (iCAC802 [35]) and *Clj* (iJL680 [45]). Exchange reactions for acetoin, acetone, 2,3-butanediol, and isopropanol, as well as secondary alcohol dehydrogenase and 2,3-butanediol-dehydrogenase reactions, were added to model iJL680 to account for the expression of genes *sadh* and *23bdh* confirmed through isopropanol and 2,3-butandeiol accumulation during fermentation and transcriptome sequencing (RNA-seq) results (48). Note that both *sadh* and *23bdh* are likely expressed at a basal level in *Clj* monoculture but are highly overexpressed in the presence of acetone and acetoin in coculture with *Cac* (48). The biomass molecular weights for each GSM model were rescaled so they were standardized to 1.0 g mmol^−1^ using the method developed by Chan et al. (62). The reaction content from nonhybrid models was used in the construction of hybrid cell models of metabolism for both *Cac* and *Clj* in proportion to their relative abundances. The iCAC802 and iJL680 reaction contents are provided in Data Set S2 in the supplemental material.

10.1128/mSystems.01325-20.2 DATA SET S2 Metabolic reaction content, flux bounds of iCAC802 and iJL680 metabolic models, and flux variability analysis results for nonhybrid and hybrid *Cac* and *Clj* models. Download Data Set S2, XLSX file, 0.4 MB. Copyright © 2021 Foster et al. 2021 Foster et al. https://creativecommons.org/licenses/by/4.0/ This content is distributed under the terms of the Creative Commons Attribution 4.0 International license.

10.1128/mSystems.01325-20.3 DATA SET S3 Growth model-predicted relative and absolute abundance of hybrid and nonhybrid *Cac* and *Clj*. Download Data Set S3, XLSX file, 0.2 MB. Copyright © 2021 Foster et al. 2021 Foster et al. https://creativecommons.org/licenses/by/4.0/ This content is distributed under the terms of the Creative Commons Attribution 4.0 International license.

10.1128/mSystems.01325-20.4 DATA SET S4 Maximum titers predicted by DMMM simulation for cell fusion case, no-cell-fusion case, and no H_2_ cross-feed case, and fermentation profiles estimated with added constraints. Download Data Set S4, XLSX file, 0.2 MB. Copyright © 2021 Foster et al. 2021 Foster et al. https://creativecommons.org/licenses/by/4.0/ This content is distributed under the terms of the Creative Commons Attribution 4.0 International license.

### Dynamic multispecies metabolic modeling.

We used the dynamic multispecies metabolic modeling framework (DMMM) (49, 63), which extends the concept of dynamic flux balance analysis (dFBA) (64) to microbial communities. DMMM links the outlined growth model with the previously mentioned nonhybrid and hybrid state GSM models in an integrative modeling framework. Twelve GSM models (replicates of the original two) were included in DMMM: a single nonhybrid model for each organism and five hybrid state models per organism corresponding to the hybrid states defined in the growth model. Rather than standard Monod growth kinetics (as was used previously [49, 63]), the hybrid state growth model developed in this study was used to determine organism/hybrid state cell abundances. To capture the effect of progressive nonnative protein dilution during hybrid cell growth within our metabolic simulations, we assign hybrid state glucose uptake efficiencies to hybrid state cells which correlate with the amount of nonnative protein contained in cells at any given hybrid state. Table 3 lists the glucose uptake efficiency for each hybrid state under the assumption that nonnative protein is halved after each doubling time. We assume homogeneous protein distribution and that glucose uptake efficiency is directly proportional to the total amount of glucose transporter proteins in a given hybrid state cell. Experimental glucose and fructose concentration curves constrained total culture substrate utilization. Substrate concentration values between experimental measurements were estimated using p-chip interpolation (60). The forward Euler method (65) has been applied to solve the DMMM problem. Flux variability analysis (FVA) has also been implemented at each time step within DMMM to predict maximum theoretical fermentation product titers and identify feasible ranges for CO_2_ and H_2_ cross-feeding (evolved from *Cac*, consumed by *Clj*) across the 33-h time span of the simulation.

**TABLE 3:** Hybrid-state glucose uptake efficiency

Organism	Hybrid state	Glucose uptake efficiency (%)	Glucose uptake (mmol gDW^−1^ h^−1^, basis = 5 mmol gDW^−1^ h^−1^ nonhybrid *Cac* glucose uptake)
*Cac*	Nonhybrid	100	5
	1	50	2.5
	2	75	3.8
	3	87.5	4.4
	4	93.8	4.7
	5	96.9	4.8
*Clj*	Nonhybrid	0	0
	1	50	2.5
	2	25	1.3
	3	12.5	0.6
	4	6.3	0.3
	5	3.1	0.2

### Data availability.

All data sets used in this study and codes for running the simulations described in this article can be downloaded from our group’s GitHub page: https://github.com/maranasgroup/DMMM. This includes all experimental data; metabolic reaction content; flux bounds of iCAC802, iJL680, hybrid *Cac*, and hybrid *Clj* metabolic models; and supplemental MATLAB and GAMS codes for running all DMMM and FVA simulations described in this article.
